# Prioritising viral hepatitis elimination to prevent hepatocellular carcinoma: A public health approach for effective preventive hepatology

**DOI:** 10.1016/j.jhepr.2025.101436

**Published:** 2025-05-02

**Authors:** Dana Ivancovsky Wajcman, Aina Nicolàs, Camila A. Picchio, Lena van Selm, Geoffrey Dusheiko, Zobair M. Younossi, John F. Dillon, Saleh A. Alqahtani, Homie Razavi, Massimo G. Colombo, Achim Kautz, Gregory J. Dore, Jeffrey V. Lazarus

**Affiliations:** 1 Barcelona Institute for Global Health (ISGlobal), Barcelona, Spain; 2 University College London, School of Medicine, London, United Kingdom; 3 Beatty Liver and Obesity Research Program, Inova Health System, Falls Church, VA, USA; 4 The Global NASH Council, Washington DC, United States; 5 Center for Outcomes Research in Liver Disease, Washington DC, USA; 6 Division of Respiratory Medicine and Gastroenterology, School of Medicine, University of Dundee, Dundee, United Kingdom; 7 Liver, Digestive, and Lifestyle Health Research Section, and Organ Transplant Centre, King Faisal Specialist Hospital and Research Centre, Riyadh, Saudi Arabia; 8 Division of Gastroenterology and Hepatology, Weill Cornell Medicine, New York, NY, USA; 9 Center for Disease Analysis Foundation, Lafayette, CO, USA; 10 San Raffaele Hospital, Liver Center, Via Olgettina, Milan, Italy; 11 Kautz^5^, Köln, Germany; 12 The Kirby Institute, UNSW Sydney, Sydney, Australia; 13 City University of New York Graduate School of Public Health and Health Policy (CUNY SPH), New York, NY, USA; 14 Faculty of Medicine and Health Sciences, University of Barcelona, Barcelona, Spain

**Keywords:** Hepatocellular carcinoma, liver cancer, marginalised populations, preventive hepatology, viral hepatitis

## Abstract

Hepatocellular carcinoma (HCC) is the most common type of primary liver cancer, accounting for about 70-80% of cases globally. The hepatitis B and C viruses (HBV and HCV) account for approximately 70% of all HCC cases worldwide, with variation across geographic regions. While progress has been made in achieving some of the World Health Organization's viral hepatitis elimination targets set for 2030, considerable action is still needed to achieve global viral hepatitis elimination. Although numerous viral hepatitis prevention strategies, including vaccination against HBV, have proven successful, considerable gaps and challenges remain in their implementation. Likewise, monitoring for additional risk factors for HCC continues to be insufficient. This is particularly important given that the burden of viral hepatitis is further compounded by the high and rising prevalence of steatotic liver disease (formerly called fatty liver disease), a growing global concern and a major HCC driver. A more comprehensive approach to HCC prevention is critical and we propose an evolving narrative which emphasises an expanded understanding of “preventive hepatology” as a framework. Preventive hepatology recognises that the growing burden of liver cancer, mainly HCC, can be effectively addressed through the prevention and treatment of viral hepatitis, with additional, targeted preventive measures being applicable for populations at risk, through screening and surveillance. A more holistic approach to HCC prevention should include primary prevention strategies for the early detection and timely treatment of viral hepatitis and steatotic liver disease. It should also include HCC surveillance among people living with chronic viral hepatitis infection, particularly those living with cirrhosis, those cured of HCV, and the management of additional risk factors associated with other HCC aetiologies.

## Graphical abstract

**Figure:**
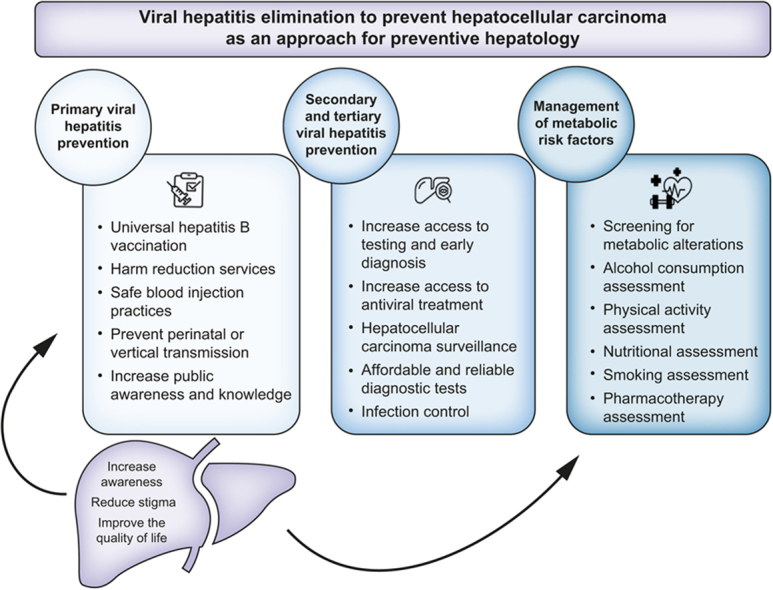

**Keypoints**

• Although integration of viral hepatitis and hepatocellular carcinoma (HCC) in care guidelines has increased over the last decade, and HCC surveillance recommendations are now included in most viral hepatitis guidelines, implementation of such practices is sub-optimal and requires further evaluation.
• Despite most HCC guidelines emphasising the importance of hepatitis B virus vaccination and hepatitis C virus treatment, more emphasis is needed on viral hepatitis case detection, early treatment initiation, and preventive treatment, to reduce the risk of transmission and HCC.
• Viral hepatitis guidelines must underscore the significance of steatotic liver disease as a risk factor for HCC and its management, via lifestyle modification, as an essential component of a comprehensive approach to preventive hepatology.
• Our new definition of “preventive hepatology” acknowledges that the growing burden of HCC can be effectively addressed through the prevention, diagnosis, and treatment of viral hepatitis, coupled with HCC monitoring strategies, and reduction of exposure to risk factors that contribute to steatotic liver disease.

## Introduction

Primary liver cancer is the third most common cause of cancer-related death worldwide.^1^ Hepatocellular carcinoma (HCC) is the main histological subtype of primary liver cancer and accounted for 70-80% of global cases between 2018-2019,^2^^,^^3^ followed by intrahepatic cholangiocarcinoma, which accounted for 15% of cases.^3^ The hepatitis B and C viruses (HBV and HCV) are the most common antecedent risk factors for HCC, accounting for approximately 70% of all cases globally.^4^ However, the burden of other aetiologies is increasing. In 2019, HBV and HCV accounted for 41% and 29% of global primary liver cancer deaths, respectively, followed by alcohol at 19%, overweight- and obesity-related liver disease, such as metabolic dysfunction-associated steatotic liver disease (MASLD, formerly called non-alcoholic fatty liver disease^5^) and metabolic dysfunction-associated steatohepatitis (MASH, formerly called non-alcoholic steatohepatitis^5^), at 7%, and other causes at 5%.^2^^,^^4^ However, between 2010-2019, HCV was associated with the lowest increase in attributable incidence of liver cancer cases (+23%), while MASH showed the fastest increase in attributable incidence of liver cancer cases and cancer deaths (+39% and +38%, respectively).^2^ Furthermore, the prevalence of chronic HBV, alcohol-related liver disease (ALD), and ALD with chronic HCV-related HCC increased 2.4-fold, 3.1-fold, and 6.4-fold, respectively.

In 2016, the World Health Organization (WHO) called for the elimination of viral hepatitis as a public health threat by 2030. In line with this, the 2022 WHO Global Health Sector Strategy (GHSS) on viral hepatitis aims to increase HBV and HCV diagnosis and treatment rates from 30% of all cases in 2020 to 90% diagnosed and, of those, 80% treated by 2030.^6^ Of the 55 countries that developed viral hepatitis national strategic action plans for 2022-2030, only 36 (65%) included HBV and HCV elimination targets, 21 (38%) had a national budget or financing plan for viral hepatitis control activities, and 18 (33%) included targets for needle and syringe programmes for people who inject drugs (PWID). To advance the global elimination goal, in 2021, the WHO introduced a standardised framework to track progress on viral hepatitis elimination.^7^ The United Nations also included combatting viral hepatitis in the Sustainable Development Goals (target 3.3), but only HBV has a clear measurement indicator.^8^

Prevention strategies play a pivotal role in viral hepatitis elimination efforts by preventing new infections, enabling timely diagnosis, and enhancing effective and timely treatment and management of infections. Viral hepatitis infection prevention can be structured into primary, secondary, and tertiary prevention, which are interconnected. Primary prevention focuses on mitigating the risks of acquiring the disease and includes advocacy and awareness, HBV vaccination, blood safety strategies, harm reduction, and safer injection and sex practices.^9^ Complementarily, secondary and tertiary prevention emphasise timely diagnosis (*e.g*. testing and treatment programmes for at-risk populations) and care to decrease the impact of ongoing disease and foster the long-term well-being of people living with the condition.^9^

Despite the promise of such strategies, in 2022, only 13.4% and 36.4% of people living with chronic HBV and HCV were diagnosed, respectively, and 2.6% and 20% of those living with HBV and HCV were treated, respectively.^9^ Prophylactic vaccination will therefore not address the current morbidity of individuals living with chronic HBV infection, as many remain unaware of their status.^6^ While no cure for chronic HBV infection is available, antiviral agents can suppress viral replication and reduce the risk of cirrhosis, HCC, and liver-related death.^10^

While global HBV vaccination has proven effective in reducing the global burden of HCC and is endorsed by viral hepatitis and liver cancer guidelines, other viral hepatitis prevention strategies have been less well adopted. In addition, there are gaps in the implementation of actions highlighted in guidelines and recommendations, particularly across those regions and countries accounting for the highest burden of disease.^9^ Herein, we review the latest strategies for preventing, treating, and managing viral hepatitis, highlighting their essential role in HCC prevention. Additionally, we underscore the importance of integrating recommendations across viral hepatitis and HCC management guidelines, and the challenges in implementing these strategies in clinical practice.

## Primary prevention of viral hepatitis

### Strategies reported in guidelines

Viral hepatitis elimination and liver cancer prevention guidelines emphasise strategies for primary prevention of HBV and HCV. In general, the following four main actions are suggested: 1) universal access to HBV vaccination; 2) strengthening the prevention of perinatal or vertical HBV transmission; 3) expanding harm reduction services for PWID and promoting safer sex practices; and 4) promoting safer healthcare practices, including universal screening of blood products and using sterilised instruments for surgery and injections.

Viral hepatitis elimination guidelines^6^^,^^11^^,^^12^ and the IARC’s (International Agency for Research on Cancer’s) world cancer report^13^ mention all of these strategies, while other cancer prevention guidelines focus mainly on HBV vaccination[14], [15], [16], [17] (Table 1). In 2019, *The Lancet Gastroenterology & Hepatology* commissioners developed additional critical recommendations for accelerating the elimination of viral hepatitis, which expanded beyond universal and birth dose HBV vaccination.^18^ These included recommendations about PWID and people experiencing incarceration, such as the provision of opioid agonist therapy and needle exchange programmes, alongside strengthening the quality of blood-transfusion services and increasing awareness around the overuse of medical injections.

**Table 1:** Summary of viral hepatitis primary prevention strategies, required actions, and implementation gaps.

Strategy	Guidelines mention this intervention		Gaps to implement	Action/policy to enhance implementation
	Viral hepatitis guidelines [reference]	Cancer prevention guidelines [reference]		
Strengthening the prevention of mother-to-child HBV transmission	AASLD-HBV,^45^ APASL-HBV,^46^ GHSS,^6^ EASL-HBV,^47^ WHO-HBV,^12^ WHO-ER^11^	IARC^13^	• Lack of policies in national immunisation programmes • Out-of-pocket payments to cover HBV birth dose • Home deliveries	• Support decentralised and community-based vaccination programmes • Promote decentralised and mobile health units to facilitate vaccine delivery in remote or rural areas • Governmental support to ensure free or affordable HBV birth doses
Universal access to HBV vaccination	AASLD-HBV,^45^ APASL-HBV,^46^ GHSS,^6^ EASL-HBV,^47^ WHO-HBV,^12^ WHO-ER^11^	AASLD-HCC,^15^ APASL-HCC,^14^ Argentina-HCC,^49^ EASL-HCC,^17^ EC,^16^ IARC^13^	Low-middle income countries: • Vaccine unavailability • Financial barriers • Home deliveries and rural health • Maintaining the cold chain High-income countries: • Vaccine hesitancy	• Awareness-raising programmes on vaccination safety and importance • Increased research and funding for the development of HCV vaccine • Financial support or subsidies
Expanding harm reduction services for PWID and promoting safe sex practices	AASLD-HCV,^51^ GHSS,^6^ EASL-HCV,^48^ WHO-HBV,^12^ WHO-ER^11^	IARC^13^	• Low adherence to screening and follow-up • Low levels of awareness of risky behaviours and practices	• Provision of an accelerated HBV vaccination schedule (0, 1, and 2 months)^27^ • Community-based and peer-led awareness and education programmes • Increasing awareness among healthcare providers
Promoting safe healthcare practices, including universal screening of blood products and using sterilised instruments for surgery and injections	APASL-HBV,^46^ GHSS,^6^ EASL-HCV,^48^ WHO-HBV,^12^ WHO-ER^11^	IARC,^13^ Argentina-HCC^49^	• Access to services provided by unqualified practitioners • Lack of awareness of the risks associated with injections or the reuse of syringes • Use of non-sterilised injections by providers believing that injections are fast-acting and more efficacious than oral drugs	• Education programmes for patients and healthcare providers • Enhancing patients' capacity to make informed decisions to effectively manage their health. • Empowering communities to advocate for safer injection practices • Implement a policy mandating using reuse prevention (RUP) injection devices in all healthcare facilities

AASLD, American Association for the Study of Liver Diseases; APASL, Asian Pacific Association for the Study of the Liver; EASL, European Association for the Study of the Liver; EC, European Commission; ER, European Region; GHSS, Global Health Sector Strategy; HBV, hepatitis B virus; HCC, hepatocellular carcinoma; HCV, hepatitis C virus; IARC, International Agency for Research on Cancer; WHO, World Health Organization.

### Examples of successful interventions

#### Uptake of HBV vaccination programmes

The successful implementation of universal HBV vaccination programmes has been key in advancing widespread coverage, following the WHO’s 1991 recommendation to include HBV vaccination in all national immunisation programmes.^19^ Global coverage increased from 1% in 1990 to 85% by 2019,^19^ yet significant disparities across geographical regions remain.

Due to the long latency period between HBV infection and the development of HCC, the impact of universal HBV vaccination coverage on HCC prevention may take many years to show clear and quantifiable evidence.^20^^,^^21^ Nonetheless, countries like China^22^ and Taiwan^23^ have already demonstrated a remarkable 70-80% and 80-92% reduction in HCC incidence and mortality, respectively, in cohorts born after the launch of vaccination programmes. Taiwan was one of the first countries to incorporate universal HBV immunisation in 1984, decreasing hepatitis B surface antigen (HBsAg) prevalence among individuals <20 years from 9.8% to 0.6% by 2004, and substantially reducing HCC incidence among all age groups.^19^^,^^24^ Data from other endemic regions, particularly across sub-Saharan Africa and the broader African diaspora, which bear a significant portion of the global HBV burden, are substantially lacking.

#### Impactful awareness campaigns

As stated in the WHO’s 2024 Global Hepatitis Report, promoting public awareness of prevention, testing, and treatment is key to viral hepatitis elimination.^9^ Public awareness campaigns worldwide have substantially contributed to the uptake of HBV primary prevention among the general population and at-risk groups. The involvement of key stakeholders, from local and regional authorities to community members, has been crucial in achieving impactful outcomes. Furthermore, the development of culturally and linguistically relevant and appropriate activities and resources has facilitated their effective uptake. These efforts have not only led to increased awareness, but also increased screening, HBV vaccination (including the birth dose), and linkage to specialist care rates, ultimately reducing the disease burden in the long term.^25^^,^^26^

#### Harm reduction services

PWID are disproportionately affected by viral hepatitis infections, particularly HCV. Harm reduction services, such as needle exchange programmes and opioid agonist therapy, have the capacity to reduce the transmission of these infections, and can be used to offer accessible and decentralised testing and treatment.^27^

### Gaps in implementing primary prevention strategies: From guidelines to reality

Despite improvements in HBV vaccination coverage and incidence targets,^6^ much remains to be done globally to reach the WHO’s viral hepatitis elimination targets. Here, we address the main gaps in implementing primary prevention strategies (Table 1).

#### Low public knowledge and awareness

Low levels of public awareness and knowledge pose a major barrier to tackling viral hepatitis, including increased exposure to risk factors and low rates of HBV vaccination, screening, and treatment.^18^^,^^28^^,^^29^ Beyond augmenting uptake of viral hepatitis services, increased awareness can contribute to mitigating stigma and discrimination against people living with viral hepatitis.^30^ Avoiding behaviours that may increase their probability of exposure (*e.g.* condomless sex, needle sharing, and unsafe healthcare practices) is the first step to avoiding infection. However, populations disproportionately affected, including PWID or migrants from high-prevalence countries, are often unaware of these risks.[31], [32], [33] Moreover, gaps in healthcare professionals’ knowledge and awareness of HBV continue to influence their practices and attitudes toward its prevention.^9^^,^^34^

#### Insufficient vaccination coverage

Perinatal or vertical HBV transmission is a major risk for increased cases of chronic HBV, as an estimated 90% of individuals infected at birth develop chronic HBV.^9^ HBV birth dose coverage and a complete infant HBV vaccination schedule are critical to prevent vertical and early childhood transmission.^7^ Despite 115 countries introducing universal HBV birth dose vaccination, wide coverage, and free access are not ensured.^9^ Additionally, the Global Alliance for Vaccines and Immunization´s vaccine investment strategy for 2021-2025 included the HBV birth dose but, partially because of the COVID-19 pandemic, available funding was paused until reassessment in 2024.^35^ This is particularly relevant given that global HBV birth dose coverage remains low (45%), with deficient coverage in the African region (18%). Africa accounts for 63% of new HBV infections, with approximately 5% of the population living with HBV.^9^ A modelling study indicated that reaching WHO 2030 HBV vaccination targets will be challenging, especially in Africa, highlighting the urgent need to enhance the care cascade.^36^

Barriers to extensive HBV birth dose administration include a lack of or limited policies in national immunisation programmes, constrained access to newborns within the first 24 h after birth, and out-of-pocket payments to cover expenses.^9^ In low-income countries, high rates of home deliveries hinder access to newborns for HBV birth dose vaccination,^37^ as a result of the physical distance to health facilities,^38^ low access to decentralised healthcare professionals, and insufficient infrastructure to store and deliver vaccinations in ambient conditions.^39^

HBV vaccine coverage among children is estimated to be 84% globally, yet geographical disparities persist, particularly in low-income countries.^9^ In regions with moderate-to-high HBV prevalence, barriers include vaccine unavailability and financial constraints.^9^^,^[40], [41], [42] In contrast, countries with accessible HBV vaccines face vaccine hesitancy, driven by a diminished perceived risk and growing concerns over vaccine safety, contributing to lower vaccination rates.^43^^,^^44^

## Secondary and tertiary prevention of viral hepatitis

### Strategies reported in guidelines

Secondary and tertiary prevention strategies play an important role in viral hepatitis prevention guidelines. Generally, guidelines strongly focus on the following strategies: 1) early screening; 2) early diagnosis and linkage to care; 3) antiviral treatment; and 4) HCC surveillance.

Viral hepatitis elimination guidelines^6^^,^^11^^,^^12^^,^[45], [46], [47], [48] and IARC^13^ mention all of these strategies, while cancer prevention guidelines[14], [15], [16], [17]^,^^49^ mainly mention antiviral treatment and HCC surveillance but omit the importance of early viral hepatitis screening, diagnosis, and linkage to care (Table 2). *The Lancet Gastroenterology & Hepatology* 2019 commissioners recommend increasing the focus on national viral hepatitis elimination progress and financing measures to provide affordable and high-quality near-care diagnostics and access to treatment for all, including vulnerable populations.^18^ Additionally, applying an equitable approach to diagnosis and treatment, and strategies to reduce stigma are also important for viral hepatitis elimination.^48^^,^^50^

**Table 2:** Summary of secondary and tertiary prevention strategies, required actions, and implementation gaps.

Strategy	Guidelines mention this intervention		Implementation gaps	Action/policy to enhance implementation
	Viral hepatitis guidelines [reference]	Cancer prevention guidelines [reference]		
Early screening for viral hepatitis	AASLD-HBV,^45^ AASLD-HCV,^51^ APASL-HBV,^46^ GHSS,^6^ EASL-HBV,^47^ WHO-HBV,^12^ WHO-ER^11^	IARC^13^	• Low levels of awareness of viral hepatitis and its risks • Insufficient decentralised services (*i.e.* community-based) • High out-of-pocket costs related to screening • Lack of testing in primary care • Lack of awareness of primary care practitioners on screening	• Promotion and investment in decentralised screening programmes • Promotion and investment in decentralised screening programmes
Early diagnosis and linkage to care	AASLD-HBV,^45^ APASL-HBV,^46^ GHSS,^6^ EASL-HBV,^47^ WHO-HBV,^12^ WHO-ER^11^	IARC^13^	• Limited availability of screening and treatment products, especially in primary care • Insufficient decentralisation of services • Limited access for rural populations • High OOP associated with treatment	• Uptake of screening opportunities in primary care and community settings to enable timely diagnosis and treatment
Antiviral treatment	AASLD-HBV,^45^ AASLD-HCV,^51^ APASL-HBV,^46^ GHSS,^6^ EASL-HBV,^47^ WHO-HBV,^12^ WHO-ER^11^	AASLD-HCC,^15^ APASL-HCC,^14^ Argentina-HCC,^49^ EASL-HCC,^17^ EC,^16^ IARC^13^	• Low adherence to treatment • Lack of comprehensive clinical research and cost-effectiveness analyses for HBV treatment	• Establish a simplified pathway for treatment to increase adherence and monitoring • Governmental support to ensure free or affordable antiviral treatment • Conduct further clinical research to assess the impact of expanded HBV treatment on HCC incidence • Perform cost-effectiveness studies to evaluate universal treatment strategies • Adopt patient-centred care approaches to improve treatment experiences • Implement the 2024 WHO HBV guidelines to expand treatment eligibility and align with updated recommendations
HCC surveillance	AASLD-HBV,^45^ AASLD-HCV,^51^ APASL-HBV,^46^ GHSS,^6^ EASL-HBV,^47^ WHO-HBV,^12^ WHO-ER^11^	AASLD-HCC,^15^ APASL-HCC,^14^ Argentina-HCC,^49^ EASL-HCC,^17^ IARC,^13^ LI-RADS^144^	• Lack of awareness and knowledge among healthcare professionals • Lack of high-quality evidence of effectiveness	• Promote educational programmes to increase awareness on HCC among healthcare providers • Promote further research on HCC surveillance cost-effectiveness • Simplified algorithms, particularly for low- and middle-income countries

AASLD, American Association for the Study of Liver Diseases; APASL, Asian Pacific Association for the Study of the Liver; EASL, European Association for the Study of the Liver; EC, European Commission; EU, European Region; GHSS, Global Health Sector Strategy; HBV, hepatitis B virus; HCC, hepatocellular carcinoma; HCV, hepatitis C virus; IARC, International Agency for Research on Cancer; LI-RADS, Liver Imaging Reporting and Data System; OOP, out-of-pocket; WHO, World Health Organization.

In terms of treatment, guidelines recommend treatment for all treatment-naïve and treatment-experienced people living with HCV, including those without significant fibrosis or cirrhosis.^48^^,^^51^ The universal duration of HCV treatment is 8-12 weeks; however, it might vary depending on prior treatment experience.^48^

### Examples of successful interventions

While global coverage of viral hepatitis screening, diagnosis, and treatment remains low,^9^ some regions and countries are successfully moving towards achieving the viral hepatitis elimination targets set to be reached by 2030.^6^ Egypt has demonstrated unprecedented success, becoming the first country to achieve WHO validation on the path to HCV elimination.^52^^,^^53^ Due to unsafe injection practices in the 1950s-1980s, Egypt’s HCV prevalence rose to 10% in 2015.^54^ Since then, the combination of political will and action, translating into the provision of universal access to free HCV screening and treatment, has resulted in an 87% and 93% rate of diagnosis and treatment/cure, respectively.^52^ Taiwan has also made remarkable progress following the launch of a national vaccination programme, which increased accessibility to treatment by approving full anti-HBV treatment coverage. HBV treatment rates thus rose, contributing to a decrease in liver-related morbidity and mortality.^55^

A critical contributor to the success of HCV treatment, and progress towards its elimination, has been the transition from genotype-specific direct-acting antivirals (DAAs) to highly effective and simplified pan-genotypic DAA regimens, largely obviating the need for prior genotyping.^56^ Additionally, the use of reflex testing strategies, whereby additional testing is performed automatically, *e.g*. for HCV RNA, HBV DNA, or anti-hepatitis D virus (HDV), following a positive anti-HCV or HBsAg test, has augmented screening and diagnosis. Reflex testing accelerates diagnosis, facilitating timely treatment initiation and minimising loss to follow-up, which is of particular concern among many disproportionately affected and underserved populations.^57^

The implementation of prevention programmes targeting groups with increased prevalence has also proven effective in increasing timely diagnosis and linkage to care. For instance, interventions including peers and decentralised provision of care enhance treatment engagement among PWID.[58], [59], [60] Decentralised care service delivery via strategies such as cultural mediators and community health workers has successfully addressed barriers faced by migrants, including those pertaining to access, culture, and language.^61^ Moreover, the provision of free or affordable services contributes to mitigating financial constraints that might impede access to care.^61^ Collectively, the implementation of these strategies has markedly increased awareness, screening, and linkage to care rates, facilitating early diagnosis and treatment.

### Gaps in implementing secondary and tertiary prevention strategies: from guidelines to reality

Antiviral treatment for chronic viral hepatitis infections mitigates the risk of disease progression, reducing the incidence of HCC.^15^ However, the uneven global distribution of diagnosis and treatment coverage represents a substantial barrier to the elimination of viral hepatitis. Certain regions are disproportionally affected by these infections and challenges in increasing diagnosis and treatment rates. In 2022, HBV diagnosis rates ranged from 26% in the Western Pacific to <3% in Southeast Asia, while HCV treatment coverage ranged from 3% in Africa to 35% in the Eastern Mediterranean.^9^

The low availability of screening and treatment products, particularly in primary care and community-based settings, underscores an insufficient decentralisation of services, hindering access to care, particularly, for example, for rural populations.^9^^,^[62], [63], [64] Economic barriers resulting from screening and treatment costs exceeding the global benchmark, coupled with inadequate domestic funding attributable to low levels of political will and engagement, and low levels of local production, further increase regional disparities.^9^^,^^62^^,^^65^ Screening and treatment rates can also be determined by sociodemographic aspects, with factors like lower socioeconomic standing, being a migrant, language barriers, and stigma potentially hindering access to and use of such services.^66^^,^^67^

DAA therapy for HCV greatly reduces HCC development risk, yet most people with HCV remain undiagnosed,^6^ including many with advanced liver disease, which puts them at an increased risk of HCC.^68^^,^^69^ A predictive algorithm demonstrated that people exhibited HCV symptoms, on average, 2-3 years before their diagnosis,^70^ indicating the need to increase disease awareness and reduce diagnostic delays. Also, despite achieving a sustained virologic response (SVR), some people living with HCV and cirrhosis remain at an increased risk of HCC.^71^^,^^72^

Despite the nearly 10-fold increase in the number of people receiving treatment for chronic HCV infection since 2015,^6^ estimates based on 2017-2019 data demonstrated that, of 45 high-income countries, only 11 were on track to eliminate HCV by 2030.^73^ This can be partially explained by the COVID-19 pandemic, which hampered HCV and HBV elimination programmes by impacting testing and treatment efforts,^74^^,^^75^ with a total of 43% of countries reporting related disruptions.^76^ A modelling study estimated that pandemic-induced HCV service interruptions in 2020 will result in 44,800 and 72,300 excess HCC cases and liver-related deaths, respectively, between 2020-2030.^77^

Gaps also exist in the implementation of effective HCC surveillance strategies, with healthcare professionals failing to initiate or maintain them, for instance, because of insufficient awareness and knowledge.^78^^,^^79^ This can also be partially explained by the lack of high-quality evidence on their effectiveness.^79^ Constraints pertaining to time, distance, and economics also impact people´s engagement with treatment and follow-up, and hinder successful surveillance.^78^

## Viral hepatitis prevention and treatment as a strategy to prevent HCC

### Integration of viral hepatitis and HCC prevention across guidelines

**Fig. 1:**
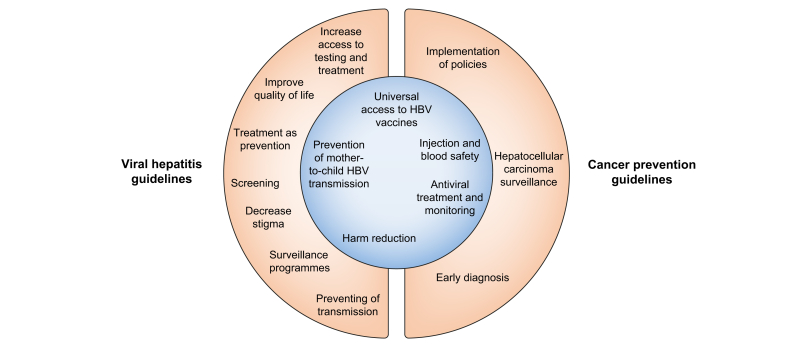
Overview of the viral hepatitis prevention recommendations outlined in clinical guidelines. The inner circle displays recommendations that are common to both viral hepatitis and cancer guidelines, whereas the outer ring displays recommendations that are mentioned only in either viral hepatitis or cancer guidelines.

In recent years, efforts to integrate viral hepatitis prevention and HCC management into health policies have intensified. For instance, the 2008-2013 WHO Global Action Plan for the prevention and control of noncommunicable diseases^80^ did not mention viral hepatitis, while the 2016 action plan for the WHO European Region included preventing HCC through HBV immunisation in its policy options for member states to implement^81^. Likewise, the 2020 WHO report on cancer includes HBV vaccination and early diagnosis and treatment of viral hepatitis as key interventions for primary cancer prevention.^82^ Viral hepatitis guidelines show a similar evolution; in the 2016-2021 WHO GHSS on viral hepatitis, the importance of defining the health burden of and providing quality treatment for HCC was mentioned, but no integrated actions were proposed,^83^ while the 2022-2030 WHO GHSS on viral hepatitis explicitly calls for strengthening the integration and linkage between viral hepatitis and liver cancer management (action 69).^6^ The 2024 WHO report on viral hepatitis also stresses the importance of integrating viral hepatitis prevention services into national cancer programmes (action 3.3).^9^ Fig. 1 summarises primary, secondary, and tertiary viral hepatitis and HCC prevention strategies mentioned in management guidelines. Integrating viral hepatitis and primary HCC prevention and management efforts is essential to minimise the substantial public health burden associated with these conditions.^6^^,^^84^^,^^85^

### HCV treatment as an HCC prevention strategy

EASL clinical recommendations on HCV treatment^48^ use the term ‘treatment as prevention’ to refer to the use of treatment to prevent onward HCV transmission, especially among PWID. This concept of treatment as prevention first became well known in the HIV field. Evidence from 2011 suggests that expansion of antiretroviral therapy provision by initiating treatment earlier, at higher CD4+ cell count cut-off points, prevented HIV transmission and could be used as a treatment and prevention strategy.^86^ Since the introduction of DAAs to treat HCV, the discussion of ‘treatment as prevention’ in the context of viral hepatitis has increased, and several modelling studies have provided supportive evidence for this approach.^87^ Empirical data on HCV treatment as prevention demonstrated a halving of HCV incidence among a large prison-based cohort following rapid DAA scale-up.^88^

In addition to preventing onward HCV transmission, achieving SVR, even among those living with advanced fibrosis or cirrhosis, reduces the chance of developing HCC and liver failure.^89^ A modelling study suggested that eliminating HCV would reduce the burden of HCC by more than 65% globally.^90^ Medical treatment with interferon (IFN) or DAAs has been demonstrated to be an effective way to reduce HCC occurrence and recurrence.^91^^,^^92^

A 2017 meta-analysis of 41 articles (n = 13,875 people) showed that HCC occurrence was low following HCV treatment resulting in SVR, with no statistical differences between treatment with IFN *vs.* DAA therapy,^93^ suggesting that antiviral treatment serves as an important strategy to prevent HCC. Recommendations reiterate the importance of curative antiviral therapy to achieve viral eradication and prevent clinical decompensation among people living with HCV who are receiving curative intent treatment for HCC.^94^ While the HCC recurrence rate was lower among people who achieved SVR in a meta-analysis,^93^ two small studies reported higher HCC recurrence rates following DAA-induced SVR.^95^^,^^96^ Given the substantial resources needed to monitor people after HCV clearance with DAAs, it is essential to identify those at high risk of HCC to optimise management.^96^

### HBV treatment as an HCC prevention strategy

HBV treatment mainly includes nucleos(t)ide analogues (NAs) and pegylated–IFN–α.^97^ Despite there being no cure for HBV, treatment with IFN or NAs has been shown to reduce HCC risk.^98^ A comparison of matched cohorts of people living with HIV and HBV, who initiated therapy upon diagnosis, and people living with HBV who were also treated showed a 60% reduction in HCC incidence among the former, possibly due to earlier treatment initiation.^99^^,^^100^ The NAs entecavir or tenofovir are the most common antiviral therapies used to treat chronic HBV infection. HIV and HBV-coinfected patients are treated with anti-retroviral regimens including either tenofovir or tenofovir alafenamide. While NA regimens show comparable safety and antiviral efficacy, their impact on HCC development has not been prospectively studied.^101^^,^^102^ Some reports have suggested that the reduction in the risk of HCC is greater with tenofovir than with entecavir, but these data are confounded by methodologic pitfalls.^103^

HBV replication is suppressed by NAs, although maintenance suppressive therapy is required. However, existing care guidelines are confounded by their complexity, so simplified principles are needed to expand treatment eligibility. The 2024 WHO HBV treatment guidelines recommend treatment for all people living with HBV DNA >2,000 IU/ml and an alanine aminotransferase (ALT) level above the upper limit of normality (ULN) (30 U/L for child and adult males and 19 U/L for child and adult females);^12^ as for adolescents, ALT levels should be above the ULN on at least two occasions within a 6 to 12-month period. While these guidelines are aimed at treatment simplification in resource limited settings, the absence of access to HBV DNA tests in these settings could hinder monitoring for advanced liver disease and HCC. Thus, guidelines recommend that in the absence of access to an HBV DNA assay, those with persistently abnormal ALT levels (defined as two ALT values above the ULN at unspecified intervals during a 6-12-month period) alone, regardless of their APRI (aspartate aminotransferase-to-platelet ratio index) score, could be treated; however, more research is needed to validate this approach. Individuals living with HBV DNA >2,000 IU/ml or an APRI score above the recently recommended WHO cut-off of 0.5, or older adults are considered at increased risk of liver disease.^9^^,^^12^ Universal treatment could be expanded to all people living with detectable HBV DNA and raised serum aminotransferases, although the long-term benefits of this approach are not well established and the number of individuals that would need to be treated to prevent one case of HCC is higher at lower HBV DNA strata, as these people are at lower risk of disease progression.^104^ Further clinical research and cost-effectiveness analyses are crucial to evaluate the potential impact of expanded HBV treatment on HCC incidence^12^. The perspectives, values, and preferences for treatment of the patient should also be taken into consideration so that care is person-centred. Of note, the 2024 WHO guidelines for the prevention, diagnosis, care, and treatment for people with chronic hepatitis B infection have not only expanded HBV treatment eligibility but also removed the term “who not to treat”.^12^

To reduce the need for lifelong treatment, functional cures, which are defined as achieving undetectable HBsAg (*i.e.* <0.05 IU/L), with or without anti-HBs seroconversion, and undetectable serum HBV DNA 6 months after stopping all treatment, are being sought.^105^ The most advanced treatments being investigated include entry inhibitors, RNA interference agents, also known as small-interfering RNAs, HBsAg assembly agents, capsid assembly modulators and immunomodulatory therapies.^106^ Broad access to therapeutic strategies with high functional cure rates is probably several years away; however, the promise of curative strategies should not slow access to treatment of hepatitis B with existing therapies.

While HBV infection poses a significant risk of HCC, coinfection with HDV is associated with an estimated two-fold higher likelihood of developing HCC compared to HBV monoinfection.[107], [108], [109] HDV depends on HBV's existence to achieve viral assembly and persistence, but the replication of HDV does not depend on HBV’s proteins or life cycle.^110^ HBV-HDV coinfection (*i.e*. simultaneous infection with HBV and HDV) or superinfection (*i.e.* HDV infection in people living with chronic HBV) is linked to accelerated liver damage and higher mortality rates compared to other chronic viral hepatitis infections, including HBV monoinfection.^108^^,^^111^ In a 2020 meta-analysis, the prevalence of HDV was 4.5% among people living with HBV (*i.e*. HBsAg+), and only 16.4% of the latter attended hepatology clinics.^112^

Governmental and non-governmental organisations must prioritise the development of cost-effective diagnostic tools for pregnant women living with HBV and individuals meeting the eligibility criteria for initiating antiviral therapy.^113^ Given the availability of generics, the absence of community access and simplified referral processes to care remain the greatest obstacles in tackling HBV.^114^ At an estimated 10%,^115^ current levels of HBV diagnosis and treatment are grossly insufficient.

### HCC surveillance

An important HCC prevention strategy is to monitor people living with viral hepatitis for HCC, considering their increased risk of advanced liver disease complications. HCC surveillance is effective when it enables earlier detection and increases the chances of curative treatment. Therefore, targeting the appropriate populations for surveillance is crucial and dependent on many variables that are beyond the scope of this review. The 2024 EASL guideline recommends a shift towards personalised surveillance based on risk factors.^17^ Below, we briefly discuss the populations among whom HCC surveillance is recommended, according to the main HCC guidelines (Table 3).

**Table 3:** Summary of hepatocellular carcinoma management guidelines surveillance recommendations.

Name of report, organisation, year [reference]	Recommended populations for regular surveillance/surveillance should be done on an individual risk stratification basis					Populations among whom surveillance is not cost-effective
	Child-Pugh A-B cirrhosis	Child-Pugh C cirrhosis	Chronic HBV without cirrhosis	Chronic HCV without cirrhosis	MASLD	
EASL Clinical Practice Guidelines: Management of HCC, EASL, 2024^17^	Recommended for any cirrhosis aetiology	Recommended only for liver transplantation candidates	Patients at intermediate or high risk of HCC (according to PAGE-B classes for Caucasians, respectively 10-17 and ≥18 score points)	HCC surveillance cannot currently be recommended for people living with chronic liver disease with advanced fibrosis (without cirrhosis), owing to insufficient evidence - no specific recommendation by aetiology		People living with Child-Pugh C cirrhosis who are not eligible for transplantation People living with a relatively high risk of death from non-HCC causes
AASLD Practice Guidance on prevention, diagnosis, and treatment of HCC, AASLD, 2023^15^	Recommended for any cirrhosis aetiology (including among people living with an active HCV infection or post-SVR attainment)	Recommended only for liver transplantation candidates	Recommended for: (a) males from an endemic country^a^ aged >40 years, (b) females from an endemic country^a^ aged >50 years, (c) people from Africa at a median age of 46 years, (d) people with a family history of HCC, (e) people with a PAGE-B score ≥10	Among people living with chronic HCV with advanced fibrosis (F3) without cirrhosis, HCC surveillance should be done on an individual risk stratification basis	Among people living with MASLD without cirrhosis, HCC surveillance should be done on an individual risk stratification basis	People living with Child-Pugh C cirrhosis who are not eligible for transplantation People with a life expectancy <1-2 years, not addressable by transplantation or other therapies People without HCV-related cirrhosis, post-SVR attainment
Argentinian clinical practice guideline for surveillance, diagnosis, staging and treatment of hepatocellular carcinoma, Argentinean Association for the Study of Liver Diseases, 2020^49^	Recommended for people living with cirrhosis and preserved liver function, irrespective of aetiology	Recommended for people living with decompensated cirrhosis listed for liver transplantation	Suggested for any person living with chronic liver disease with F >2			Not applicable
			Recommended for: (a) people living with a family history of HCC, (b) people post viral eradication of HBV with F >2, (c) people with a PAGE-B score ≥10	Recommended for people living with fibrosis (F >2), even after SVR attainment	Not applicable	
Asian Pacific clinical practice guidelines on the management of hepatocellular carcinoma: a 2017 update, APASL, 2017^14^	Recommended for any cirrhosis aetiology	Recommended only for liver transplantation candidates	Recommended for carriers who are: (a) Asian males aged >40 years, (b) Asian females aged >50 years, (c) Africans aged >20 years, (d) people with a family history of HCC	Evidence is uncertain for people living with advanced fibrosis, even after SVR attainment	Evidence is uncertain for people living with non-cirrhosis MASLD	People living with Child-Pugh C cirrhosis who are not eligible for transplantation

AASLD, American Association for the Study of Liver Diseases; APASL, Asian Pacific Association for the Study of the Liver; EASL, European Association for the Study of the Liver; HBV, hepatitis B virus; HCC, hepatocellular carcinoma; HCV, hepatitis C virus; MASLD, metabolic dysfunction-associated steatotic liver disease; SVR, sustained virologic response.

a Endemic country as defined by AASLD hepatitis B virus guidance.

HCC surveillance is strongly recommended and cost-effective among people living with cirrhosis (Child-Pugh A-B) of any aetiology,^15^^,^^17^^,^^49^ based on the annual HCC incidence of ≥1-1.5% within this population.^15^ As for people living with Child-Pugh C, surveillance is only recommended for those who are waiting for liver transplantation, since early-stage HCC can lead to being given a higher priority on the transplantation list.^15^^,^^17^^,^^49^ Among people living with a non-cirrhotic chronic HBV infection, surveillance can be considered cost-effective for at-risk groups with an annual HCC incidence of ≥0.2% (Table 3).^15^

As for HCV, HCC surveillance is recommended among those who have cirrhosis, even after achieving SVR, as they remain at an increased risk of HCC for up to 10 years after achieving SVR.^15^ This recommendation is supported by the EASL position paper on clinical follow-up after HCV cure, which recommends that all patients with pre-SVR compensated advanced chronic liver disease (F3 or F4 METAVIR) who achieve SVR with DAA therapy undergo lifelong HCC surveillance with ultrasound screening every 6 months.^94^ Conversely, for people living with HCV who achieve SVR in the absence of cirrhosis, guidelines^15^ and a modelling study^116^ suggest that surveillance is not cost-effective. There is insufficient evidence to support routine HCC surveillance among people living with HCV and advanced fibrosis (≥F3).^17^ This population might benefit from risk stratification for future HCC surveillance.^15^

Among people living with MASLD, although nearly one-fourth of MASLD-related HCC cases occur without cirrhosis, the annual HCC incidence is estimated at 0.008 per 1,000 person-years, meaning that routine surveillance is not cost-effective.^15^ As per different cohorts, liver fibrosis, along with its progression, is the primary determinant of liver-related events across the entire spectrum of steatotic liver disease (SLD).^117^ There is insufficient evidence to recommend HCC surveillance among individuals living with non-cirrhotic MASLD. However, individuals within this population, particularly those living with advanced fibrosis or additional metabolic risk factors, may benefit from personalised risk stratification assessment for future HCC surveillance.^15^

In terms of methodology, HCC surveillance should be performed via ultrasound and alpha-fetoprotein (AFP) at semi-annual intervals, approximately every 6 months.^15^^,^^17^ Two emerging HCC surveillance strategies are the liquid biopsy and the GALAD biomarker panel, which incorporates a person´s gender, age, *Lens culinaris* lectin binding subfraction of AFP, AFP, and des-gamma-carboxy prothrombin.^15^

Notably, HCC surveillance in high-income countries improves rates of diagnosis at an early stage, but 95% of people in sub-Saharan Africa present with advanced or terminal disease. For surveillance programmes to be effective, a means of implementing treatment for early HCC is required. Unfortunately, the scarcity of resources in sub-Saharan Africa will limit the likelihood of beneficial secondary prevention until opportunities for early-stage HCC treatment with curative intent are increased.^118^ Thus, measures are required to improve surveillance for early-stage HCC while improving the possibility of interventions for early HCC – *i.e.* creating centres for transarterial treatments, ablation, or hepatic resection to improve the outlook for individuals with small HCC amenable to these measures.

## Preventive hepatology

**Fig. 2:**
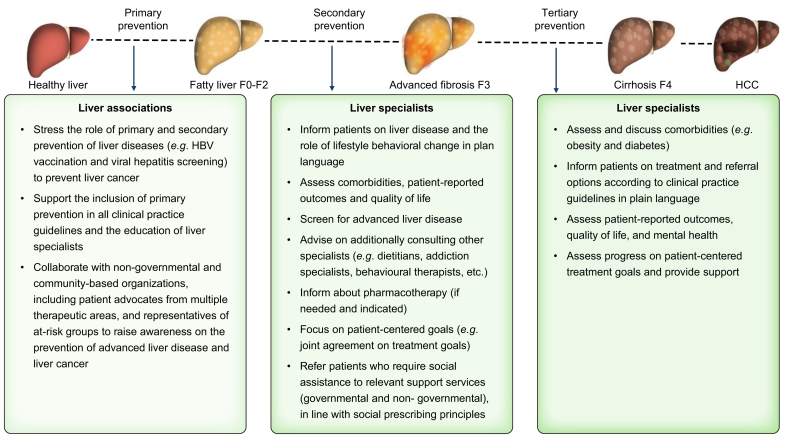
‘Preventive Hepatology’ framework for preventing new-onset liver diseases and their deterioration. Examples of actions liver associations and specialists can take at each prevention level for effective preventive hepatology. HBV, hepatitis B virus; HCC, hepatocellular carcinoma.

The term “preventative hepatology”, coined in the literature in 2008 by Hirschfield *et al.*, focuses on preventive strategies to optimise liver health in people living with chronic liver disease via timely interventions and avoiding treatment side effects.^89^ The emphasis of the original definition is on secondary and tertiary prevention; we suggest broadening this term to a “preventive hepatology” that focuses on a wider range of strategies. Our proposed expansion includes primary prevention of new-onset liver diseases, including HBV and HCV, in addition to the prevention of disease deterioration and progression, alongside management of other risk factors, such as metabolic factors that contribute to SLD. Preventive hepatology should also involve recognising and addressing the full range of determinants that impact health. Patterns of alcohol consumption and overweight, obesity, and type 2 diabetes, which are risk factors for HCC,^119^ are intertwined with social, commercial, and legal determinants and vary among different populations globally.^50^ Assessing the determinants of health associated with HCC at societal, health system, and clinical levels can inform targeted interventions for specific groups at higher risk.^120^ Liver associations can play an important role in raising awareness about them, as was done, for example, in “The EASL–*Lancet* Liver Commission”.^50^ In Fig. 2, we outline some of the actions liver associations and specialists can take to enhance preventive hepatology, including “treatment as prevention” along with primary prevention.

### Management and treatment of additional risk factors

MASLD-related HCC predominantly affects older individuals. It is diagnosed at a later stage, is well known to develop in the absence of cirrhosis, and has poorer survival compared to viral hepatitis-related HCC.^121^ Although MASLD prevalence is generally low among those living with chronic HBV,^122^ it increases their risk of HCC. A similar risk is observed in individuals living with chronic HCV,^123^ even if they are virally suppressed.[124], [125], [126]

The combination of high alcohol intake and viral hepatitis often results in more severe hepatic damage; however, the mechanisms for this are not fully understood. While it is estimated that 20-30% of those living with chronic viral hepatitis will develop cirrhosis,^6^ a 2020 meta-analysis estimated that the prevalence of cirrhosis among people living with chronic HBV who consumed alcohol excessively was higher, at 32%.^127^

HCC risk factors, such as exposure to aflatoxin B1 (AFB1), a potent hepatocarcinogen, are also distributed differently across the globe. Epidemiologic studies have substantiated a strong correlation between dietary levels of AFB1 and the incidence of HCC in individuals living with HBV in sub-Saharan Africa. Staple foods, including maize and ground nuts, are liable to such contamination, resulting in chronic hazardous exposure.^128^^,^^129^ Integrated agronomic approaches to reduce crop AFB1 contamination are urgently needed to mitigate this risk.

### Metabolic comorbidity assessment in guidelines

Maintaining a healthy lifestyle, including having a balanced diet and performing regular physical activity, is a key determinant in preventing cancer, including HCC. Several strategies to enable healthy lifestyles are mentioned in the Word Cancer Report,^13^ including mandatory nutrition labelling, reducing carcinogenic food content, limiting unhealthy food marketing for children, increasing taxes on sugar, including soft drinks, and alcohol, promoting sustainable transport, and encouraging exercise, including daily physical activity.

However, HCC management guidelines do not address lifestyle in a comprehensive manner. For instance, HCC guidelines highlight the importance of physical activity in managing metabolic diseases^14^ and as a primary prevention strategy for HCC,^15^ but do not provide specific recommendations. Aerobic physical activity has been shown to moderately reduce liver steatosis (*i.e.* a 2-4% absolute reduction). Any type of physical activity provides essential extrahepatic benefits, including improved body composition, cardiorespiratory fitness, and health-related quality of life.^130^^,^^131^ In addition, although smoking is mentioned as a potential risk factor for HCC incidence, guidelines do not explicitly recommend smoking cessation.^14^^,^^15^

Additionally, HCC guidelines[13], [14], [15]^,^^17^^,^^49^ highlight that, based on low-to-moderate evidence, coffee consumption is associated with a reduced risk of HCC in individuals living with chronic liver disease and should be considered among this population. Coffee contains numerous biologically active compounds, with phenolic acids and caffeine potentially inhibiting HCC cell proliferation, suppressing HCC progression, and reducing oxidative damage to hepatocytes. Additionally, coffee may mitigate insulin resistance and improve glucose metabolism, potentially slowing liver disease progression.^132^ In addition, observational studies have associated medications such as aspirin, statins, and metformin with decreased odds of HCC development. However, as these medications carry risks of toxicity and adverse events, their use solely for HCC prevention is not recommended.^15^^,^^17^

Viral hepatitis guidelines recommend assessing alcohol consumption rates and advising abstinence among everyone living with active and chronic HBV/HCV infection to reduce the risk of HCC.[45], [46], [47], [48] However, additional aspects of lifestyle such as physical activity or diet are only partially covered (Table 4). The 2022-2030 WHO GHSS on viral hepatitis addresses the importance of managing metabolic risk factors, such as obesity, diabetes, and hypertension (action 67),^6^ but does not supply practical recommendations on how to do this.

**Table 4:** Summary of global health agencies' recommendations on metabolic assessment and lifestyle modifications for patients with viral hepatitis.

Title, organisation, year [reference]	Assessment of metabolic comorbidities and/or alcohol use	Recommendations for lifestyle changes
Guidelines for the prevention, diagnosis, care and treatment for people with chronic hepatitis B infection WHO, 2024^12^	Comorbidities (*e.g*. diabetes, metabolic dysfunction-associated steatotic liver disease) and alcohol consumption should be assessed before initiating antiviral therapy	Counselling on lifestyle modification should be given as part of the initial assessment. Lifestyle modification includes weight reduction, regular exercise, avoiding alcohol, sugar-sweetened beverages, and ultra-processed foods
Hepatitis C Guidance 2023 Update: American Association for the Study of Liver Diseases– Infectious Diseases Society of America Recommendations for Testing, Managing, and Treating Hepatitis C Virus Infection AASLD, 2023^51^	Addressing hazardous alcohol use	N/A
Global health sector strategies on, respectively, HIV, viral hepatitis, and sexually transmitted infections for the period 2022-2030 WHO, 2022^6^	Assessing alcohol intake is recommended for all people with chronic viral hepatitis infection	Behavioural interventions to cease or reduce alcohol intake
EASL recommendations on treatment of hepatitis C: Final update of the series EASL, 2020^48^	The contribution of comorbidities (*e.g.* alcohol use, renal dysfunction, diabetes, obesity, steatotic liver disease) to the progression of liver disease must be evaluated before and after drug therapy	Alcohol consumption should be assessed and quantified, with specific counselling given
Update on Prevention, Diagnosis, and Treatment of Chronic Hepatitis B: AASLD 2018 Hepatitis B Guidance AASLD, 2018^45^	Assess comorbidities (*e.g.* heavy alcohol consumption or MASLD) that increase the risk of HCC	Optimisation of body weight and treatment of metabolic complications, including diabetes and dyslipidaemia. Abstinence or only limited use of alcohol is recommended in those infected with HBV
EASL 2017 Clinical Practice Guidelines on the management of hepatitis B virus infection EASL, 2017^47^	Assess for comorbidities, including MASLD and MASH, before treatment	N/A
Action plan for the health sector response to viral hepatitis in the WHO European Region WHO regional office for Europe, 2017^11^	Assess alcohol intake levels for all people with a chronic viral hepatitis infection	Behavioural alcohol-reduction interventions for those people with moderate-to-high alcohol intake
Asian Pacific clinical practice guidelines on the management of hepatitis B: a 2015 update APASL, 2016^46^	Assess for comorbidities such as alcohol use, obesity, diabetes and metabolic syndrome	Patients with chronic HBV infection should be counselled regarding lifestyle modifications. Counsel to avoid excessive alcohol use

AASLD, American Association for the Study of Liver Diseases; APASL, Asian Pacific Association for the Study of the Liver; EASL, European Association for the Study of the Liver; HBV, hepatitis B virus; HCC, hepatocellular carcinoma; HIV, Human Immunodeficiency Virus; MASLD, metabolic dysfunction-associated steatotic liver disease; MASH, metabolic dysfunction-associated steatohepatitis; WHO, World Health Organization.

Evidence on the potential protective role of weight loss and a healthy diet in the prevention of severe liver disease and HCC is based on observational studies.[133], [134], [135], [136] Lifestyle research is challenging because of methodological limitations including the complex nature of the exposure (*e.g.* physical activity, diet), the difficulty in defining and measuring the exposure well, and the length of the follow-up period.[137], [138], [139] Thus, even though a healthy lifestyle and weight reduction are important, the level of evidence around these might hinder related recommendations from being widely adopted in guidelines.

Addressing overweight, obesity, SLD, type 2 diabetes, and other metabolic alterations constitutes a fundamental component of preventive hepatology and a pivotal strategy to reduce HCC rates in general, and among people living with HBV and HCV in particular.^140^^,^^141^ A crucial advance towards holistic preventive hepatology involves implementing health-promotion policies that effectively support individual behavioural modifications. For instance, taxing alcohol and unhealthy food like sugar-sweetened beverages, alongside subsidies for healthy food, would help to regulate the affordability and availability of such items and reduce disease burden.^142^ Such shifts are fundamental in accomplishing proactive hepatological care.^143^ Viral hepatitis guidelines must thus underscore the importance of diagnosing and managing metabolic comorbidities as part of the treatment of HBV and HCV.

## Conclusions

The field of hepatology should more explicitly recognise and draw attention to the importance of viral hepatitis prevention strategies, and timely diagnosis and expanded treatment, to reduce the burden of advanced liver disease, including HCC. This evolving narrative should include an expanded understanding of preventive hepatology as a framework for such a holistic, multi-stakeholder approach. Preventive hepatology recognises that the high and growing burden of HCC should be addressed through viral hepatitis prevention, in addition to early diagnosis and treatment. Preventive hepatology should include effective primary prevention strategies for viral hepatitis, such as universal HBV vaccination, alongside secondary and tertiary prevention, such as treatment of people living with a chronic viral hepatitis infection and HCC surveillance among people living with HBV and HCV. Other HCC aetiologies must also be addressed through the prevention and management of additional risk factors, such as overweight, obesity, poor diet quality, lack of physical activity, and alcohol consumption. Despite the evidence, and at a time when global viral hepatitis elimination efforts have stagnated, there remains little focus on viral hepatitis management as an important aspect of HCC prevention.

## Abbreviations

AASLD, American Association for the Study of Liver Diseases; AFB1, aflatoxin B1; AFP, alpha-fetoprotein; ALD, alcohol-related liver disease; ALT, alanine aminotransferase; APASL, Asian Pacific Association for the Study of the Liver; DAA, direct-acting antiviral; EASL, European Association for the Study of the Liver; GHSS, global health sector strategy; HBsAg, hepatitis B surface antigen; HBV, hepatitis B virus; HCC, hepatocellular carcinoma; HCV, hepatitis C virus; HDV, hepatitis D virus; IFN, interferon; MASH, metabolic dysfunction-associated steatohepatitis; MASLD, metabolic dysfunction-associated steatotic liver disease; NA, nucleos(t)ide analogue; PWID, people who inject drugs; SLD, steatotic liver disease; SVR, sustained virologic response; ULN, upper limit of normal; WHO, World Health Organization.

## Financial support

The authors did not receive any financial support to produce this manuscript.

## Authors’ contributions

JVL conceived of the idea. DIW, CAP, and LvS drafted the first draft of the paper with JVL. GD, ZMY, JFD, SAA, HR, MGC, AK aand GD made substantial contributions to the critical review, editing, and revision of the manuscript. DIW, AN, and JVL led the revision. All authors approved the final version of this manuscript.

## Conflict of interest

CAP acknowledges consultancy fees from Roche Diagnostics, outside this work. GD acknowledges participation in safety monitoring boards for Aligos Therapeutics, Janssen, Glaxo Smith Kline, Arbutus, Myr GMBH/Gilead Sciences, Vir, Precision Biosciences and advisory boards for Antios and Gilead Sciences; he is also an advisor for the National Medical Research Council of Singapore and an unpaid consultant for the EU Horizon project Heimholtz Zentrum Muenchen Deutsches Forschungzszentrum fuer Gesundheit und Umwelt and the A-Tango European Commission grant, outside of this work. ZMY acknowledges consulting fees from Gilead, Intercept, Siemens, Novo Nordisk, Madrigal, Merck, Quest, and Bristol Myers Squibb, outside of this work. JFD acknowledges institutional grants and personal speaker fees from AbbVie, Gilead Sciences, MSD, and Roche, outside of this work. HR acknowledges membership in advisory boards for Gilead, AbbVie, Abbott, Merck, Janssen, Roche, and VBI Vaccines (all proceeds were donated to the Center for Disease Analysis Foundation) and institutional grants from Gilead, Assembly Biosciences, AbbVie, Boehringer Ingelheim, Intercept, Merck, Novartis, Pfizer, and Roche, outside of this work. MGC acknowledges membership in advisory boards for Target HCC, Exelixis, Galapagos, and Agios, outside of this work. GJD acknowledges research support from Gilead and AbbVie, outside this work. JVL acknowledges grants to ISGlobal from AbbVie, Boehringer Ingelheim, Echosens, Gilead Sciences, Madrigal, Moderna, MSD, Novo Nordisk, Pfizer, and Roche Diagnostics, consulting fees from Echosens, GSK, NovoVax, Novo Nordisk, Pfizer, and Prosciento, and payment or honoraria for lectures from AbbVie, Echosens, Gilead Sciences, Janssen, Moderna, MSD, Novo Nordisk, and Pfizer, outside of this work. DIW, AN, LvS, SAA, and AK have no potential conflicts of interest to disclose.

Please refer to the accompanying ICMJE disclosure forms for further details.
